# Similarity of activated sludge and treated wastewater with special reference to nitrifiers and their seasonal variability

**DOI:** 10.1038/s41598-025-34503-4

**Published:** 2026-01-10

**Authors:** Magdalena Domańska, Magdalena Kuśnierz, Sylwia Charazińska, Jan Gawor, Joanna Kamińska

**Affiliations:** 1 Institute of Environmental Engineering, Wrocław University of Environmental and Life Sciences, Pl. Grunwaldzki 24, 50-363 Wroclaw, Poland; 2 DNA Sequencing and Oligonucleotide Synthesis Laboratory, Institute of Biochemistry and Biophysics Polish Academy of Sciences, 02-106 Warszawa, Poland; 3 Department of Applied Mathematics, Wrocław University of Environmental and Life Sciences, 50-375 Wroclaw, Poland

**Keywords:** Biological techniques, Biotechnology, Environmental sciences, Microbiology

## Abstract

While the research into the activated sludge (AS) microbiome using next-generation sequencing (NGS) are increasingly being published, they still apply to a few wastewater treatment plants (WWTPs). Little information can be found in the scientific literature on the microbial composition of treated wastewater (TW) and bacterial activity in real WWTPs facilities. In this study, AS and TW are compared in terms of their physico-chemical parameters and bacterial community composition, with particular emphasis on nitrifying bacteria, based on the results from 16S rRNA amplicon sequencing using DNA and complementary DNA (cDNA) data and fluorescence in situ hybridization (FISH). Using a 24-h composite sample of TW rather than a grab sample yields highly representative microbial community profiles. The research revealed a decrease in biodiversity in months with lower sludge temperatures both in AS and TW. Differences among groups of nitrifying bacteria were observed during the monitoring of WWTP, suggesting that their presence in TW may not be a random occurrence but could serve as an indicator of physicochemical and operational changes within biological reactors.

**Supplementary Information:**

The online version contains supplementary material available at 10.1038/s41598-025-34503-4.

## Introduction

Ongoing urbanization and industrialization, are increasing the municipal and industrial wastewater, placing an ever-increasing burden on wastewater treatment plants (WWTPs). Nutrients and organic load are the main pollutants in wastewater, one of the key ones being nitrogen of which the global increase in wastewater is estimated to more than double between the years 2000 and 2050^1^. In order to protect aquatic ecosystems, special emphasis is placed on the proper operation of the various processes at WWTPs. Control of the quality of treated wastewater (TW) is currently based on the monitoring of nitrogen and phosphorus compounds, organic matter, as well as suspended solids. Biological removal of nitrogen and its compounds is one of the crucial processes carried out at WWTPs. Conventionally, this process involves two stages: nitrification and denitrification. The nitrification process consists a two-stage oxidation of ammonia to nitrate. Initially, ammonia is oxidized to nitrite by ammonia-oxidizing bacteria (AOB) or archaea (AOA), and then nitrite is oxidized to nitrate by nitrite-oxidizing bacteria (NOB)^2^. However, the nitrification process often fails under conditions that are unfavorable for bacterial growth. Consequently, efforts are being made to better understand the mechanisms operating within activated sludge flocs and the release of biomass into receiving water bodies. Moreover, the ongoing revision of the European Union directive on municipal wastewater treatment (2024/3019) places greater emphasis on incorporating new quality indicators, including microbiological parameters, into monitoring programs.

The structure of microbial communities changes under the influence of deterministic and stochastic factors. In the literature, opinions vary as to which factors dominate. A study by Sun et al. (2021) confirmed that of the deterministic factors, homogeneous selection had the greatest influence, and among the random factors, drift^3^. A study of 1,200 activated sludge samples collected from 269 wastewater treatment plants worldwide confirmed the importance of the structure of microbial communities in activated sludge (AS). Despite their high diversity, AS contained a small, globally shared core bacterial community of 28 operational taxonomic units (OTUs) that was strongly associated with system performance. Microbial community was influenced by dispersal and drift processes and, a lesser extent, environmental factors: temperature, biochemical oxygen demand (BOD) and chemical oxygen demand (COD). Among these, the main parameters were temperature and BOD in the influent, which were relevant to the conditions studied and could influence the processes shaping the structure of microbial communities^4^. Nevertheless, the temperature has a very strong influence on the nitrification process, and the bacterial community structure shifts less significantly in winter periods^5^. This is particularly noticeable at WWTPs located in areas where temperatures drop below 10ºC^6–8^. Temperatures around 25ºC result in the best AOB growth, although nitrification activity can be seen even at temperatures as low as 5ºC^9^. Kim et al. (2021) observed that, although the sum of the relative abundances of *Nitrospira* spp. and *Nitrotoga* spp. remained stable over time, the abundance ratio of these two organisms varied substantially. From January to May, the population of *Nitrospira* spp. gradually decreased in certain reactors. At the same time *Nitrotoga* spp. followed the opposite pattern with a sharp increase in abundance in the cold months between January and March^10^. Temperature is one of the factors influencing the breakdown of the nitrification process due to the fact that some microorganisms like *Nitrospira* are more sensitive in winter to random conditions^8^. Other factors that can negatively affect the process include pH, dissolved oxygen concentration, and chemical oxygen demand value^11^. At low dissolved oxygen concentrations, the metabolic activity of AOB can be inhibited, leading to a decrease in the efficiency of the ammonia removal process^12^.

The ability to characterize the microbial community composition in environmental samples, including those from WWTPs, has significantly improved with the development of advanced molecular biology techniques, particularly those based on short-read 16S rRNA gene sequencing. While this approach does not directly measure microbial function, it provides important insights into the microbial populations associated with key treatment processes such as AS dynamics^13^. Through molecular biology, it has been shown that some species of *Nitrospira*, which are considered NOB, perform full nitrification^14^. Despite many achievements in this field, the influence of the variables that determine the nitrification process is still unclear.

Although the genetic material in the form of DNA is stable and can survive in the environment for a very long time, it is not possible to distinguish between live and dead bacteria based only on it^15^. An analysis that potentially indicates the microbial activity is one based on ribosomal RNA (rRNA). However, many studies have indicated that in microbial communities, an accurate comparison to determine which microorganisms are viable cannot be confirmed by one method only^16,17^. Among the many reasons is that rRNA can remain long after the microorganism has died (i.e. is no longer active)^18^. Despite these limitations, rRNA is widely used to study the active microorganisms in environmental samples. Research showed that viable bacterial biomass contained 30–47% active bacteria^19^.

To evaluate the nitrification process, AS collected from the nitrification chamber is the one mostly used, but the microbial community composition of raw wastewater flowing into the WWTP is also analysed^20^. There is a lack of information in the literature on the suspended solids that leaves the WWTP with the TW in the form of microflocs. Furthermore, the mechanisms explaining the susceptibility of the sludge fraction to dispersion and detachment from the flocs are poorly understood^21^. Information is also lacking in the literature on whether the presence of bacteria in the flocs present in the effluent is coincidental, related to sludge leaching due to higher flows, or may reflect nitrification problems. This has not been analyzed before. If the two environments are sufficiently similar, monitoring of wastewater quality at WWTPs can be supplemented by monitoring the treated wastewater in order to detect bacteria responsible for nutrient reduction processes and respond earlier to changes in the system by modifying the treatment plant’s technological parameters, e.g., sludge retention time (SRT).

Research in the literature indicates that sludge flocs present in the effluent can exhibit both structural characteristics related to microbial composition or EPS and functional characteristics similar to those of flocs from AS. However, these flocs are arranged differently. Sears et al. (2006) indicated that low density flocs constitute about 2% of the total biomass, making them recognizable in the activated sludge structure and potentially transported into the outflow^22^. In addition, Li et al. (2020) and Ekholm et al. (2024) have shown that low-density flocs are biologically active and can be carried with the effluent, thereby maintaining functionality and metabolic activity^23,24^. The susceptibility of sludge in bioreactors to floc detachment is being intensively studied although it remains poorly understood. Studies on the mechanism of flocculation have shown that complete floc disintegration never occurs^25^. According to Morgan-Sagastume et al. (2008), about 60–70% of bacterial cells are associated with microcolonies and small flocs in suspension, whereas suspended biomass is in a dynamic equilibrium with the weakly flocculated sludge particles^26^. The authors showed that groups of bacteria, depending on their quantity and activity level, are more or less susceptible to detachment and suspension^26^. The study by Kalinowska (2022)^27^ confirmed our earlier assumptions that during colder periods, when nitrification problems occur, additional smaller bacterial structures in smaller flocs enter the outflow^25–29^.

This article aimed to demonstrate the comparison of two environments (activated sludge and treated wastewater) and identify which nitrifying bacteria end up in the effluent. Until now, activated sludge and suspended solids from the outflow have not been compared in terms of nitrification problems, which is an original contribution of this research. In order to comprehensively address this hypothesis, we conducted a field study with the overall objective of investigating the diversity of bacterial populations of AS and TW by the 16S rRNA amplicon sequencing method after isolating DNA and RNA. To meet the main objective, the specific objectives consisted of microscopic analysis of the AS and TW, analysis of bacteria by FISH, and testing of the physical and chemical parameters. The results were then subjected to a statistical analysis.

### Methodology

#### Wastewater treatment plant characteristic

The system under study is a full-scale WWTP in the region of Lower Silesia, Poland, that treats municipal wastewater with maximum hourly supply about 350 m^3^/h. The biological stage of wastewater treatment consists of a continuous-flow system including: one pre-denitrification tank, two dephosphatation tanks, three denitrification tanks, two nitrification tanks (bioreactors), and two secondary sedimentation tanks. Polyaluminum chloride is used to support the phosphorus removal process. Bioreactors are a core component operated under the integrated fixed-film activated sludge (IFAS) technology. Both WWTP’s bioreactors are equipped with textile biofilm fibers (Cleartec BioCurlz, Jäger, Germany), providing additional surface for the biofilm growth. Detailed specifications of the bioreactors are presented in Table 1.

**Table 1:** Operating conditions of the WWTP bioreactors.

Parameter	Values
Total volume of nitrification tanks	2860 m^3^
Maximum hourly supply	350 m^3^/h
Mean sludge age	10 days
Mean hydraulic retention time	8 h
Mean biomass concentration	6 g/l
Setpoint oxygen concentration	2 mg/l
Sludge loading rate	0.2 kg COD/kg MLSS

Chemical oxygen demand (COD). Mixed liquor suspended solids (MLSS).

#### Sampling

Two types of samples were collected: activated sludge (AS) taken directly from the activated sludge chamber (ASC), and treated wastewater (TW) taken from the outflow of the treatment plant, where a sampler pumped a defined volume of TW into the tank over a 24-h period (composite sample). One liter of each sample were transferred to the laboratory within one hour. No disinfection was applied to the wastewater prior to sampling. AS and TW samples were collected for physico-chemical analysis and fluorescence in situ hybridization (FISH) analyses. Samples for 16S rRNA amplicon sequencing were transported under refrigeration conditions to the DNA Sequencing and Oligonucleotide Synthesis Laboratory. Samples were collected six times during the year, every two months from December 2022. They were first collected 19 months after IFAS technology was implemented.

### FISH and quantitative analysis

Samples of AS and TW were fixed as soon as they were brought to the laboratory up to 1 h after sampling. The TW required repeated centrifugation to obtain enough sludge for fixation. Fixation with 4% paraformaldehyde (PFA) at 4 °C overnight was performed. The next morning, the sample was centrifuged three times. Then, the filtrate was decanted and supplemented with pure ethanol. The next steps of the procedure were performed based on Amman’s protocol^30^. Ntspa712 and Ntoga122 probes were used along with competitors to identify most members of the phylum Nitrospirae and the genus *Nitrotoga*, respectively^14,31^. *Nitrosomonas* were identified using the NSO1225 probe, which was designed to target Betaproteobacterial ammonia-oxidizing bacteria^14^. Fluorescent dye 6FAM was used to visualize the bacteria. A general probe (EUB I, II and III) was also used to identify all bacteria. The ROX dye was used for these probes. For the quantitative analysis, 15 images of AS and TW were stained with specific probe and DAPI reagent (VECTASHIELD Mounting Medium with DAPI reagent, Vector lab, part no: H-1200–10), and then the percentage ratio of the specific probe area to DAPI was calculated. The methodology is described in detail in the article by Domańska and Kamińska (2022)^32^. Results are based on calculated medians and percentiles of 25–75%, as defined in Statistica 13.3. More than 400 images were used for the analysis. The study was carried out on a Nikon Eclipse Ni-E C2 confocal microscope equipped with UV-2A, B-2A, and G-2A filters (with excitation (EX) wavelengths of 330–380, 450–490, and 510–560 nm, respectively) and Nis-Elements AR 4.30 software.

### 16S rRNA amplicon sequencing

The total environmental DNA from the sample of interest was isolated using Environmental DNA/RNA Extraction Kit (Eurx, Gdańsk, Poland) following the manufacturer’s instructions. This reagent set enables the isolation of DNA from material such as soil, slurry or sediment. For each sample, 50 mL of AS and TW was centrifuged, and 200 mg of the resulting pellet was collected for DNA extraction**.** 200 mg of sample was weighted for processing. Samples were first disrupted by pulverization with 0.4–0.6 mm diameter glass beads (Sartorius AG, Göttingen, Germany) using the TissueLyser apparatus (Qiagen, Hilden, Germany). After isolating DNA and RNA, quality was checked by running the sample on 1% agarose gel, while template quantity was measured by fluorimetry using Qubit 2.0 and High Sensitivity Picogreen reagents (Thermo Fisher Scientific, Sunnyvale, CA, USA). The quality of the isolated RNA was further improved by the use of the Clean-up RNA Concentrator kit (A&A Biotechnology, Gdańsk, Poland), which included a DNase-based DNA removal step. To ensure that the DNA was completely removed from the RNA samples, a control PCR reaction was set up, with the RNA samples serving as a template and *Escherichia coli* Top10 (Thermo Fisher Scientific, Sunnyvale, CA, USA) genomic DNA serving as a positive control. The primers used were gene-specific primers: 16S_V3-F and 16S_V4-R, which cover positions 341-357F and 785-805R, respectively, according to *E. coli* 16S rRNA gene reference sequence^33^. PCR products were checked on 1% agarose gel. The reverse transcription was carried out with the use of RevertAid First Strand cDNA Synthesis Kit (Thermo Fisher Scientific, Sunnyvale, CA, USA) containing random hexamer primers according to the manufacturer’s instructions. The obtained cDNA samples were stored in −20°C until further analysis. Amplification of the conserved bacterial 16SrRNA gene fragment covering V3 and V4 regions was done for DNA and cDNA samples in triplicate by means of the gene-specific primers extended on 5’ end with Nextera adaptors^33^ (Illumina, Sand Diego, USA). A total of 10 ng of DNA or cDNA from each sample was used as the template for PCR.

The obtained amplicons of the size c.a. 450bp were checked on 1% agarose gel and purified by Ampure XP magnetic beads (Beckman Coulter, Brea CA, USA). Amplicon libraries were pooled in equimolar ratio and indexed according to the Nextera indexing strategy by PCR (Illumina, San Diego, USA). Sample indexing allowed for pooling of amplicons for sequencing run and further extraction of the sample sequence reads from large batch of sequencing results data. 16S amplicons were sequenced on MiSeq sequencer in the DNA Sequencing and Oligonuleotide Synthesis Laboratory IBB PAS in paired end mode using 600 cycle v3 chemistry kit (Illumina, San Diego, USA).

The obtained Illumina reads were quality checked using the FastQC v.0.12.1 software (www.bioinformatics.babraham.ac.uk/projects/fastqc^34^). Raw sequencing reads were cleaned, aligned, and classified automatically by the EzBioCloud platform using the PKSSU4.0 database^35^ statistical analysis and visualization were performed using R v.4.3.3 packages: microbiome v.1.31.0 (https://microbiome.github.io/tutorials/), ggplot2 v.3.5.2 (https://ggplot2.tidyverse.org/), and Ampvis2 v.2.8.9^36^. Rarefaction analysis was performed to assess sampling depth and ensure that observed microbial diversity was not biased by differences in sequencing depth. The phyloseq v.1.22.3 (https://joey711.github.io/phyloseq/) package in R was used to randomly subsample the OTU table at various depths, and alpha diversity was calculated at each level using the Chao1 richness estimator and the exponential of the Shannon index (exp(H’))^37^. Additional indices (ACE, Jackknife, Shannon and Simpson) are provided in the supplementary materials (Table S3). The data for analysis was obtained in the form of relative abundance, % (sometimes referred to as “abundance” in the text).

To account for the compositional nature of wastewater microbiome data of top 60 most variable genera and domain- specific taxa of interest (nitrifiers), the raw genus-level count tables were subjected to a Centered Log-Ratio (CLR) transformation. The data manipulation and analysis was done in base R v.4.3.3 using tidyverse tools (readr v.2.1.5, dplyr v. 1.1.4, tibble v.3.3.0 and tidyr v.1.3.1). The transformed data were then clustered to identify patterns of bacterial community structure, and the results were visualized as a heatmap using ComplexHeatmap v.2.18 package. ΔCLR was computed as the difference between CLR(TW) and CLR(AS) per genus, and genera were clustered (Ward.D2) across months. These clusters capture sets of taxa that shift coherently between conditions. In parallel, a comparison was made of AS vs. TW trajectories per genus (Spearman ρ and temporal slopes). Genera with ρ > > 0 and similar slopes move in parallel under both conditions. Positive mean ΔCLR indicates relative enrichment in TW, whereas negative indicates enrichment in AS.

The Wilcoxon signed-rank test, a nonparametric paired test, was used to compare abundance of bacteria in AS and TW across the same sampling months. Due to the sample size and the resulting difficulty in verifying the normal distribution assumption for the variable, both parametric (Pearson) and non-parametric (Spearman) correlation coefficients were calculated.

## Results and discussion

### Suspended solids analysis

**Fig. 1:**
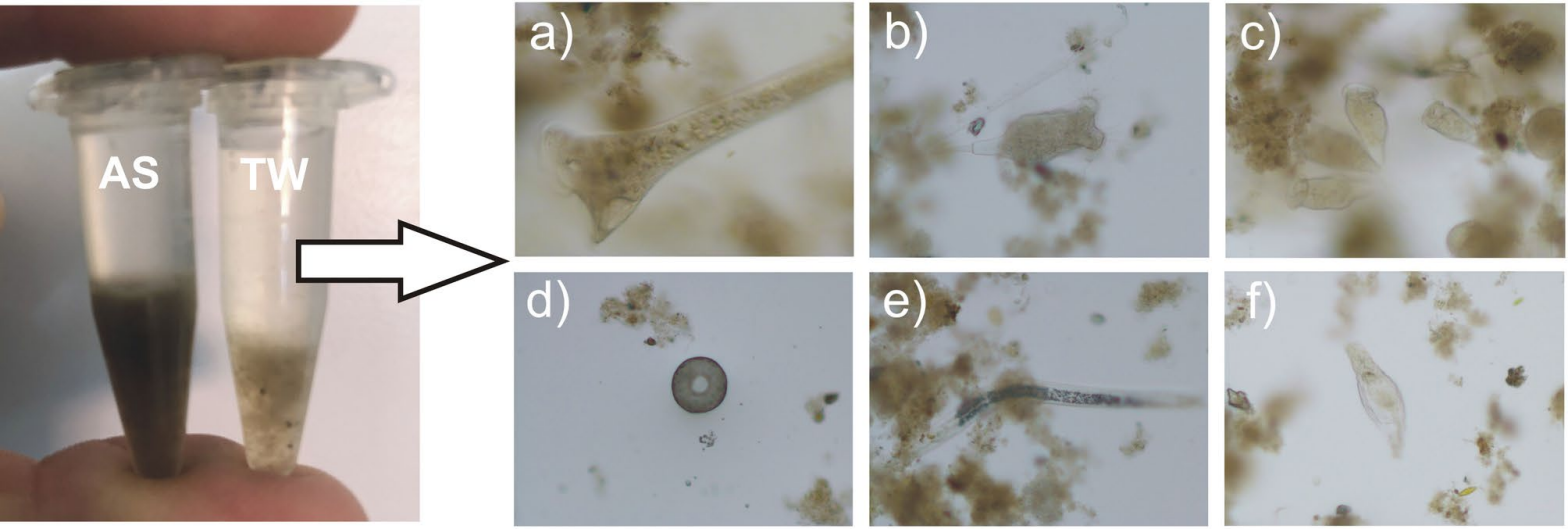
Example of an activated sludge (AS) and a suspended solids from treated wastewater (TW) in eppendorf tubes after centrifugation and the results of observations for TW : (**a**) *Stentor* sp.; (**b**) *Tokophrya* sp.; (**c**) settled ciliates; (**d**) amoeba; (**e**) nematode; (**f**) rotifer.

Of the WWTP under study, comprehensive microscopic observations of the activated sludge were conducted for a period of six months since the introduction of the IFAS technology. The findings from these observations are detailed in^38^. Current studies have confirmed that in the TW over a year, with monthly sampling, the same microorganisms were observed as those present in the AS (Figure 1).

Besides typical representatives of the AS such as settled ciliates, nematodes, rotifers, and amoebas, species like *Stentor* and *Nais elinguis* were also identified during certain periods. Confocal microscopy has also shown adhesion of bacteria variously distributed inside AS flocs and attached with varied strength. Microscopic observations led researchers to explore potential insights into the processes at the WWTP, hidden within the sludge flocs discharged with the TW.

The research conducted by Kuśnierz and Wiercik (2016) on the structure of sludge in various WWTPs also supports the study of sludge from effluent. It was recognized that the samples of TW and AS collected at the same location were characterized by similar particle size distribution^39^. It has also been proven that the flocs in bioreactors exhibited a compact structure with a high degree of compaction, while the majority of particles forming the AS were fine and small in mass^40^. Exactly this type of flocs, with lower density, were identified in the outflow from different WWTPs^29^. This may suggest that the released flocs will retain similar structural and functional properties to the larger aggregates formed during the treatment process.

**Fig. 2:**
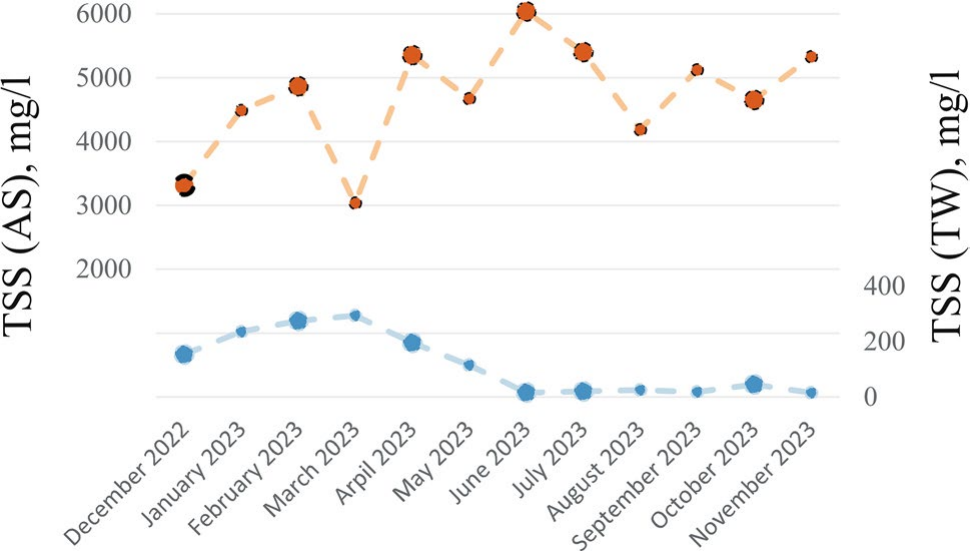
Total suspended solids (TSS) in activated sludge (AS) and treated wastewater (TW) during research period.

The amount of suspended solids in treated wastewater most often depends on the operation process, wastewater flow, and the influence of rainwater. Even in well-functioning WWTPs, complete removal of microorganisms in the effluent is not possible. After sedimentation, approximately 25 mg/l of total suspended solids (TSS) typically remains in the TW, which is well below the TSS concentration of 3–6 g/l in AS. The research showed that despite the low TSS concentration, residual biomass necessary to identify microorganisms was always contained in the TW (Figure 2). This is also confirmed by the results of the studies by Begmatov et al. (2024) and Nataraja et al. (2024)^41,42^.

### Physical and chemical parameters

**Fig. 3:**
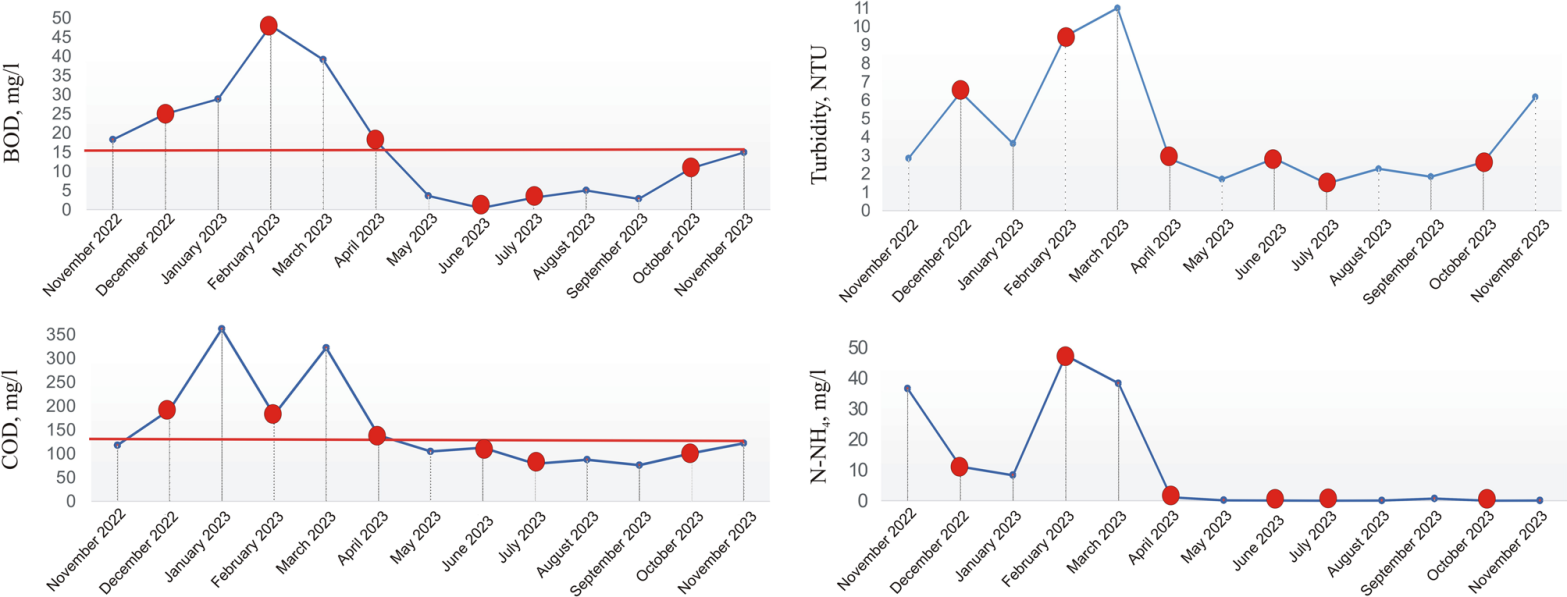
Changes in BOD, COD, Turbidity, and ammonia nitrogen (N-NH_4_) in treated wastewater (TW). Red dots mark the sampling points for both physico-chemical and microbiological analyses. The red line indicates the permissible concentration in TW according to Polish law (125 mg/l for COD and 15mg/l for BOD).

**Fig. 4:**
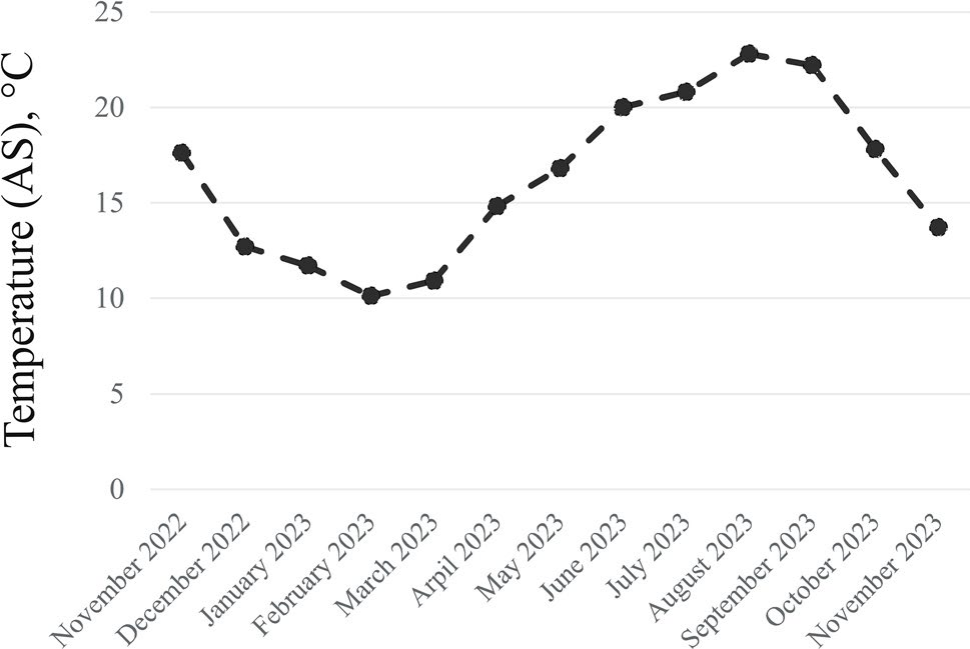
Temperature of activated sludge (AS) during research period.

The physical and chemical parameters were examined once a month. The results presented in this paper are for the period from November 2022 to November 2023. Microbiological tests were performed every other month, from December 2022 to October 2023, and the dates of collection are indicated in the graph with a red dot (Fig. 3). The research were conducted during the period when the treatment plant had problems with nitrification (elevated values of ammonium ions). In addition, elevated values of biological oxygen demand (BOD) and chemical oxygen demand (COD) at the outflow were observed, as well as an increase in turbidity during the period from November 2022 to April 2023 when there is a decrease in air temperature in Poland. The AS temperature was under 15 °C from December to April (Fig. 4).

**Fig. 5:**
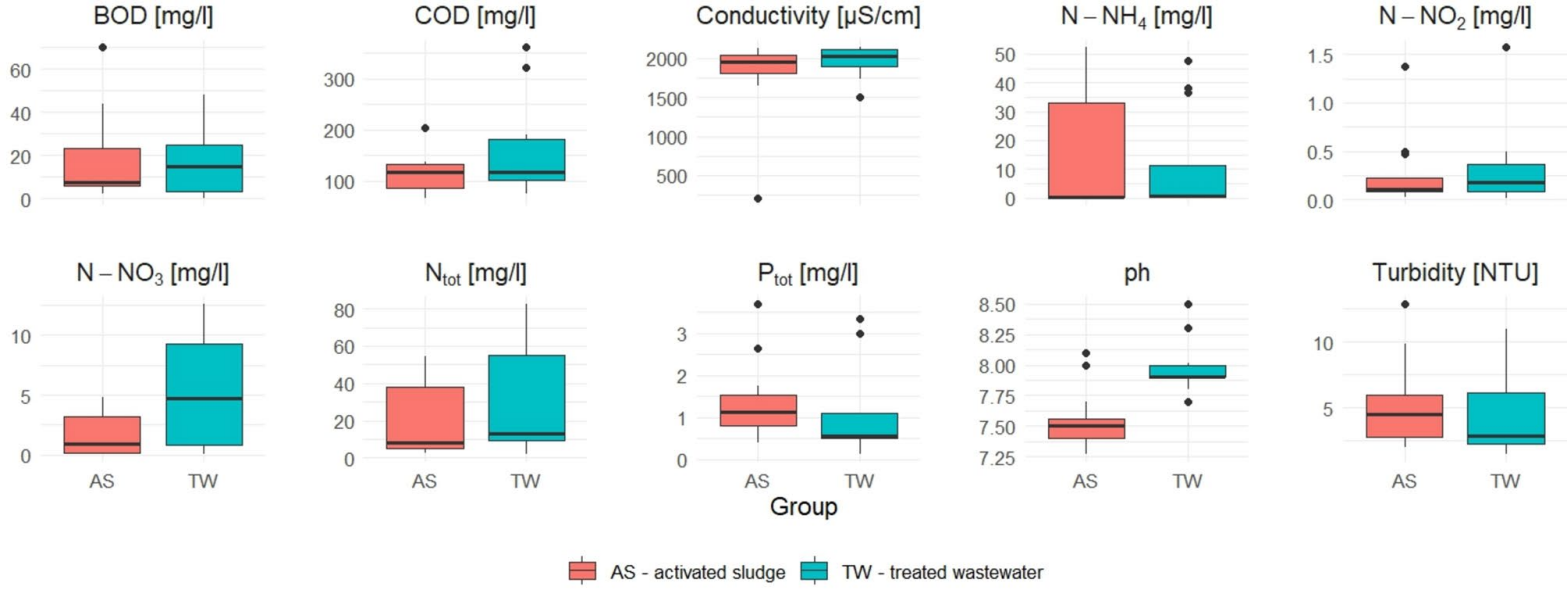
Box-plots (Q1, Me, Q3) for physical and chemical parameters in activated sludge (AS) and treated wastewater (TW) considering 13 results from November 2022 to November 2023. Dots denote outliers (Me ± 1.5·IQR) (IQR—inter-quartile range (Q3-Q1)).

Other physical and chemical parameters are presented as box-plots to compare conditions in the AS chamber and in the TW (Fig. 5).

Due to the nitrification breakdown, higher values of BOD, COD and ammonia nitrogen in the effluent were observed during the period of lower temperatures (Fig. 3). The studied parameters showed no significant differences between the values for AS and those obtained in the TW. The Student’s t-test revealed exceptions for nitrates and pH, with p-values of 0.0199 and 0.0002, respectively. Although nitrite concentrations in the TW did not exceed typical concentrations for WWTPs, they were higher compared to AS (Fig. 4). This is probably due to incomplete denitrification in the reactor or the fact that the nitrification product (NO₃⁻) accumulates mainly in the liquid phase, from which it is discharged to the TW^43^. The pH showed a wide variation. For the AS, the median for pH values was 7.5, while in the effluent it was 7.9. Although nitrifying bacteria are resistant to pH changes, individual species have different preferences for optimal growth parameters. The literature reports that for both *Nitrobacter* and *Nitrospira* the optimal pH is in the range of 8.0–8.3 for nitrite oxidation, whereas *Nitrospira* dominates under low ammonium and nitrite conditions due to its lower nitrite half-saturation constant and inhibition threshold concentrations for free ammonia and free nitrous acid^44^. For *Nitrotoga* determined the optimum occurring at pH 7.3, with dominance over *Nitrospira* at pH 7.4^45^. The lower pH of 6.4 favoured *Nitrospira*. Acidic pH demonstrated the effectiveness of *Nitrosomonas*, which can carry out autotrophic nitrification at low pH, including the confirmed effectiveness of high-rate nitrification at pH as low as 3.2^46^.

### 16S rRNA amplicon sequencing

#### Alpha diversity

**Fig. 6:**
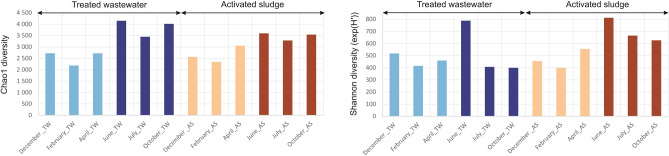
Alpha diversity indexes (Shannon exp(H′) and Chao1) were calculated using OTU abundance data obtained from the EzBioCloud database for samples of activated sludge (AS) and treated wastewater (TW) at the species level.

The microbial community of AS and TW in WWTP was investigated by 16S rRNA gene amplification and DNA sequencing (Table S1, supplementary material). In total, 1,309,386 high quality filtered reads of the 16S rRNA gene were obtained on the DNA matrix and 1,296,762 on the cDNA matrix (Table S2, supplementary material). Bacterial diversity in individual environmental samples was determined using alpha and beta diversity indexes. Alpha diversity is defined as the mean species diversity in a rather local scale^47^. In the case of Alpha diversity, the higher the value, the higher the diversity^48^. The alpha diversity indexes are shown in Fig. 6. For both the Chao1 and Shannon indexes, the highest diversity was obtained in June for the AS at 3598.63 and 788.40, and for the TW at 4148.71 and 812.41, respectively. For the Chao1 index, species richness for the AS and TW was very similar for months with lower temperatures (December, February, April), while the TW showed a higher diversity in months with higher temperatures (June, July, October) (Fig. 4). Higher Shannon diversity in TW samples (exp(H′) = 382.9 ± 57.0) compared with AS samples (exp(H′) = 252.2 ± 17.0) has also been reported in other studies^49^. For the Shannon exp(H′), a higher biodiversity was observed in the TW (versus AS) only for the winter months of December and February (Fig. 6). Other alpha diversity indices (ACE, Chao1, and Jackknife) confirmed increased species richness in the December TW sample. The lack of variation in the Simpson index suggests that dominant species remained unchanged (Table S3, supplementary material). The results indicate that the decrease in AS biodiversity may be related to problems at the WWTP, and in particular to the nitrification process. This hypothesis is supported by operational data showing increased TW NH₄⁺ concentrations during lower-temperature months (Fig. 3), which coincided with a drop in wastewater temperature (Fig. 4). This is consistent with impaired nitrification performance, as well as the results of other researchers. The results of Wagner and Loy (2002) indicated that a decrease in the biodiversity of functional groups may contribute to the system being more susceptible to a process breakdown^50^. Other research showed that the bottom sediments of a river receiving wastewater from different WWTPs contained more nitrate-oxidizing bacteria^51^. Wang et al. (2016) analysed samples taken from municipal WWTPs during summer and winter periods to show differences in the microbial community’s diversity in AS at different temperatures^52^. The Shannon diversity index was higher in summer and decreased during winter. In studies conducted on four different biological wastewater treatment systems (both domestic and industrial), the highest diversity was observed in summer, when wastewater temperatures reached approximately 36–38ºC. The lowest diversity levels occurred in spring, with moderate temperatures of around 22–25ºC, and in winter, when wastewater temperatures dropped to 14–16ºC^5^. Nevertheless, the decline in biodiversity may also indicate an increase in the prevalence of a select few dominant species.

### Beta diversity

**Fig. 7:**
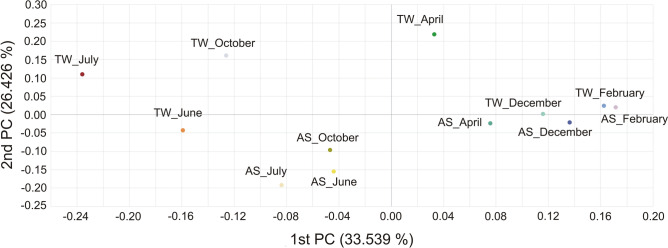
PCoA plot using unweighted Unifrac distance of the bacterial composition in the activated sludge (AS) and treated wastewater (TW) at the species level.

The diversity between microbial communities in different habitats is represented as beta diversity^53^, in this case, between treated wastewater (TW) and activated sludge (AS). The data are presented in Fig. 7 as principal coordinate analyses (PCoA) based on weighted UniFrac distances^54^.

Condensing the data on AS and TW quality into two factors retains 60% of the original information while enabling a more streamlined interpretation. The first principal coordinate (1st PC) contains one-third (33.5%) of the information about the quality of the analysed samples. This coordinate differentiates samples by temperature. Samples collected during colder months (February, December, April) tend to cluster on the right-hand side of PC1, whereas samples from warmer months (June, July, October) cluster on the left. This pattern suggests temporal structuring of the microbial community, although PC1 itself represent the dominant gradient in community composition. At the same time, it takes higher values for TW (−0.236 to −0.126) than for AS (−0.083 to −0.044). Therefore, the 1 st PC value makes it possible to determine approximately the temperature conditions. The 2nd PC usually takes on positive values for TW, but negative values for AS. This means that the greater the distance of the 2nd PC value from zero, the greater the efficiency of correctly indicating the sampling source (TW or AS). The lack of correct assignment of the PC2 value to the sampling location at values close to zero is due to the fact that the 2nd PC only explains 25% of the variability in material diversity. There is more variability in samples by wastewater temperature than by sampling source (AS-TW). This may also be related to changes in total suspended solids (TSS) in the TW, which usually depend on WWTP operating conditions, increased rainfall and temperature. Lower temperatures usually lead to a decline of the sedimentation process and an increase in runoff of suspended solids. While there was no relationship between temperature and TSS in AS, higher TSS values were observed in the TW during lower temperatures from December to April (Fig. 2).

A cluster analysis of the 60 most dynamic genera revealed variations in bacterial abundance in TW compared to AS over time. As shown in Figure S1 (supplementary material), a higher proportional abundance of bacteria was observed in the TW than in the AS during months with higher temperatures (June, July, and October). However, nitrifying bacteria are not the most abundant in AS. Therefore, the analysis was limited to bacteria associated with nitrifiers at specific phylogenetic levels in order to better understand the dynamics of these communities.

### Variability at the phylum and class levels

The Wilcoxon test revealed no statistically significant difference in bacteria abundance between AS and TW at the 0.05 level. However, a significant correlation was observed, suggesting non-random trends. The limited number of samples makes statistical assessment difficult and does not necessarily reflect an absence of significant patterns. The results of the Pearson and Spearman correlation tests showed a strong similarity between the AS and TW samples at the phylum level of 0.98 and 0.93, respectively. Considering a decrease in biodiversity during the winter months, the authors aimed to verify how these changes occur at different phylogenetic levels.

**Fig. 8:**
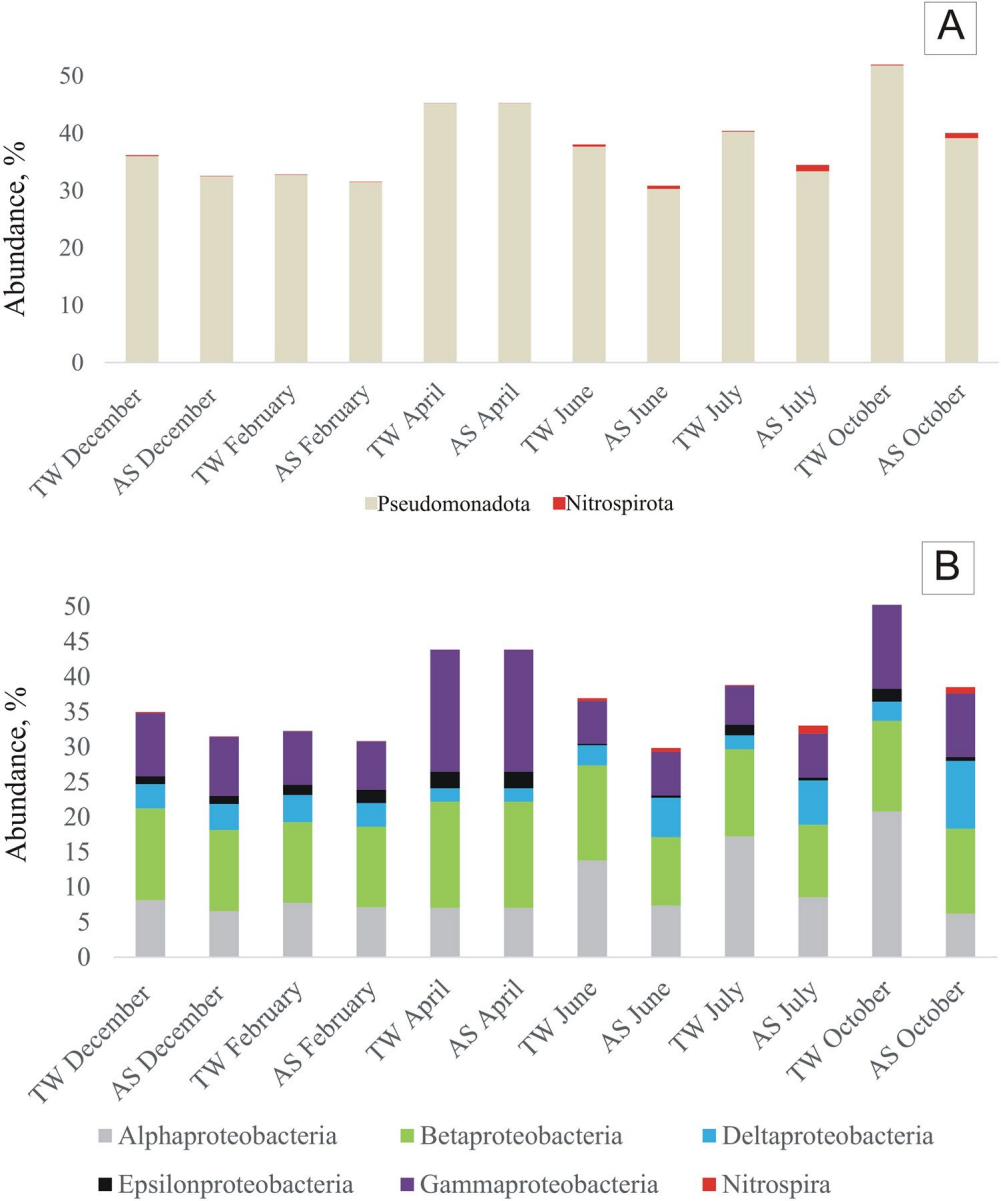
Variability of Pseudomonadota and Nitrospirota phylum (**A**) as well as proteobacterial classes (**B**) in the activated sludge (AS) and treated wastewater (TW).

The bacteria responsible for the nitrification process belong to the phylum Pseudomonadota (previously known as Proteobacteria), the class Betaproteobacteria (*Nitrosomonas*, *Nitrotoga*) and the phylum Nitrospirota. Pseudomonadota mostly predominate in the AS of Polish WWTP^55^. Analysing the samples taken over the year, more Pseudomonadota were found in the TW than in the AS except in April when they were equal (Fig. 8). Comparing samples taken in different seasons, there is a marked decline in the winter months (December, February). Phylum Nitrospirota dominated the months with higher temperatures, with the maximum abundance in July of slightly more than 1%. They were usually more abundant in AS than in TW, except from December. At the class level, the differences between the samples for months with lower temperatures (December, February, April) were less than the differences observed between AS and TW in warmer months. A similar phenomenon at the phylum level was observed by Zhang et al. (2018)^5^. The abundance of Alphaproteobacteria in treated wastewater increased noticeably in October. Higher abundances were also observed in June and July. Klausen et al. (2004) suggested that bacteria from the Beta-, Gamma-, and Deltaproteobacteria classes form stronger microcolonies^56^, while Schmid et al. (2003) noted more Betaproteobacteria in the supernatant^55^. The conducted experiments did not confirm these relationships^57^.

### Variability at the genus level

**Fig. 9:**
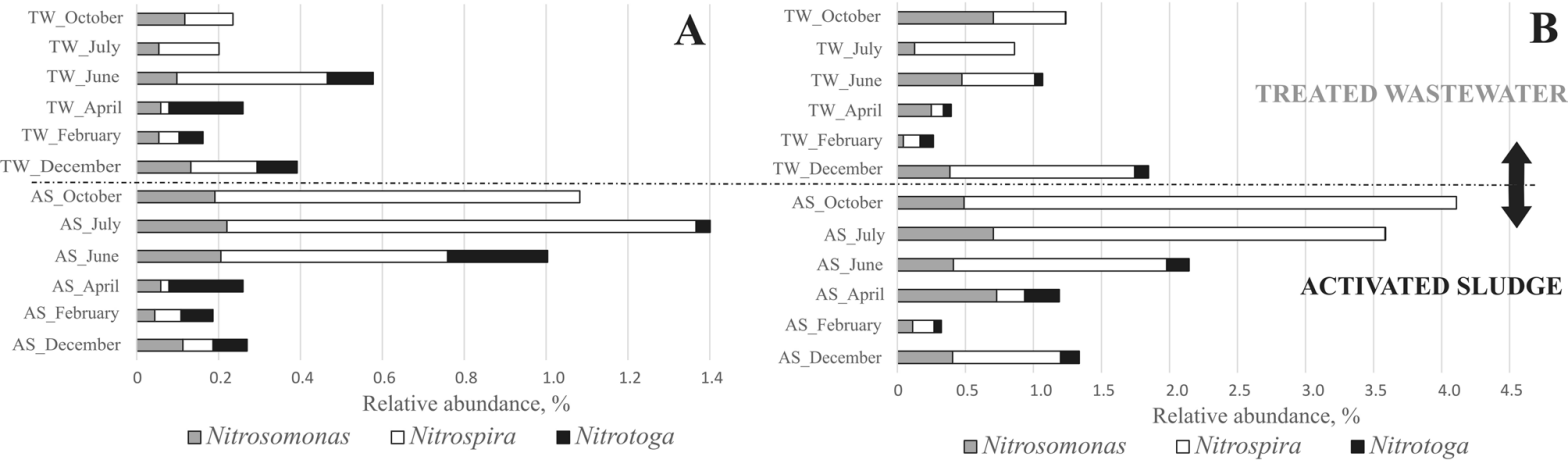
Variability of nitrifying bacteria (at genus level) in the activated sludge (AS) and treated wastewater (TW) based on (**A**) DNA and (**B**) cDNA.

When genus level was considered, a high level of similarity between AS and TW was still shown. The Pearson and Spearman correlations were similar, at 0.74 and 0.76, respectively. Results from 16S rRNA amplicon sequencing using both DNA and complementary DNA (cDNA) matrix data were used to compare the results. At the genus level, analysis of nitrifying bacteria (*Nitrosomonas*, *Nitrospira*, and *Nitrotoga*) revealed that all bacteria were present in the TW (Fig. 9).

In the case of the AS, it was clearly observed that in June, July, and October, when the temperature of the activated sludge was 20; 22.8; 17.8ºC, respectively, sum of nitrifying bacteria were much more abundant than in the months when the temperature was lower in December (12.7ºC), February (10.1ºC), and April (14.8ºC), for both DNA and cDNA (Fig. 9a, b). The percentage abundance of *Nitrosomonas* was particularly high in the AS in June, July, and October (for DNA) and in addition April (for cDNA). *Nitrotoga* bacteria was not identified in the AS in October (for DNA) and July or October (for cDNA). *Nitrospira* was present in all samples, and its abundance was higher for warmer months. In the TW, the situation was quite different, and there was no longer a significant seasonality. For the cDNA results, the TW reflected the situation in the AS except for December when more of these bacteria were also observed in the TW (Fig. 9B). The dynamics of nitrifying bacteria changes over time were also supported by cluster analysis (supplementary material, Fig. S1b). This confirmed previous results using quantitative FISH analysis that an excessive outflow of nitrifiers was observed during periods of low temperatures.

The results revealed the similarity between DNA and cDNA results, which may indicate that the most of identified bacteria were viable. In addition, elevated concentrations of BOD, COD, turbidity, and nitrification problems were observed from November 2022 to April 2023 (Figure 5). The research did not confirm findings by Morgan-Sagastume et al. (2008)^21^ that the dispersed biomass in AS has a different composition from the microbial community released from the floc. In a study conducted by Zhang et al. (2011) at 6 WWTPs, there were more *Nitrosomonas* than *Nitrospira*^58^. Their study confirmed a higher proportion of *Nitrospira* bacteria in the AS, which may suggest its lower sensitivity than *Nitrosomonas*. This is also confirmed by a study by Dionisi et al. (2002) where the authors showed that a reduction in solid retention time (SRT) caused a greater decrease in *Nitrosomonas oligotropha* than *Nitrospira*^59^.

### 16S rRNA amplicon sequencing vs FISH

**Fig. 10:**
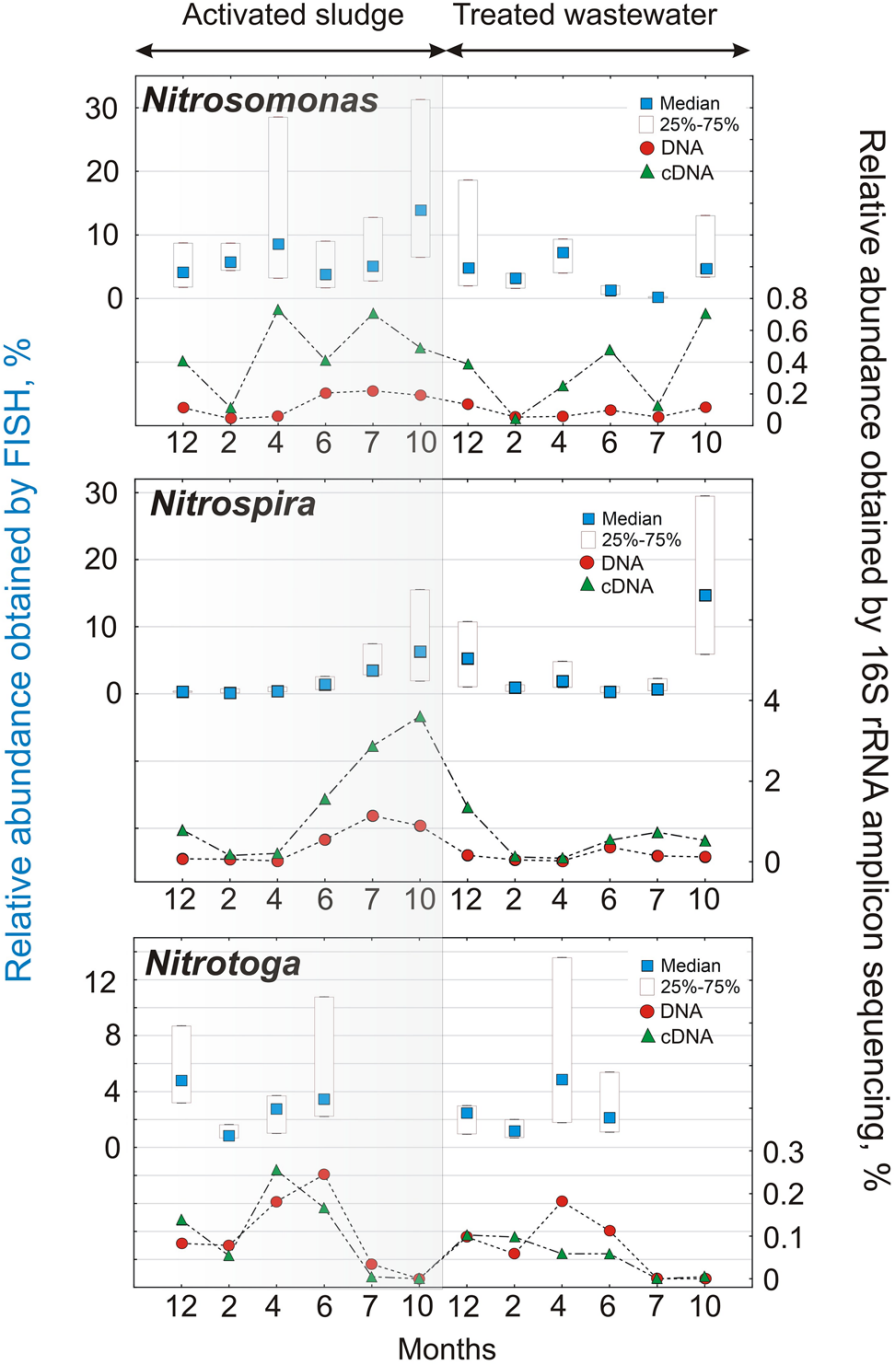
Bacteria abundance in the activated sludge (AS) and treated wastewater (TW) obtained by FISH and 16S rRNA amplicon sequencing based on DNA and cDNA in the following months (12 – December, 2—February, 4 – April, 6 – June, 7-July, 10 – October).

The FISH method is quite suitable for the identification of nitrifying bacteria, but especially for visualizing their spatial distribution with respect to other bacteria forming consortia that live in symbiosis. It can also be used for the quantitative assessment of bacteria^60^. In order to confirm the results obtained about the variability of nitrifying bacteria, the results of 16S rRNA amplicon sequencing were compared with the results of the quantitative FISH method (Fig. 10).

FISH analysis using the NSO1225 probe showed a higher abundance of these bacteria in April and October in the AS and in the TW in December, February, April, and October. 16S rRNA amplicon sequencing showed a higher presence of these bacteria in the AS during months with higher temperatures. In the TW, the results indicated a higher presence of *Nitrosomonas* bacteria in December and October. Physical and chemical research indicated that nitrification problems were observed from November 2022.

FISH analysis using the Ntspa712 probe showed an increased quantity of these bacteria in months with higher temperatures (June, July, October), consistent with the 16S rRNA amplicon sequencing results. In the outflow, a high increase in Nitrospira was observed in December and October, which matched the 16S rRNA based amplicon sequencing results. The relatively lower Ntspa/DAPI ratios in the AS during the cold months also suggested that potentially less active cells were present in the sludge. Morgan-Sagastume et al. (2008) indicated that cells from the loosely bound fraction of the activated sludge appeared to be potentially less active than cells forming medium-sized flocs of sludge^21^. According to the study, bacteria that are released into the TW were active. FISH analysis using the Ntoga122 probe did not identify these bacteria during the months of July and October in both AS and TW.

During the winter months, similar amounts of *Nitrotoga* bacteria were identified in the AS and TW, but in April there was more *Nitrotoga* in the TW than in the AS. The DNA-based analysis noted that there was a higher abundance of *Nitrotoga* in the TW than in the AS in December, while the abundances were the same in April. Nevertheless, more *Nitrotoga* than *Nitrospira* were identified in the winter months. *Nitrotoga’s* particular predisposition to grow at low temperatures was described in the work of Keuter et al. (2022)^61^. The results obtained suggest that the increased amounts of *Nitrosomonas*, *Nitrospira,* and *Nitrobacter* bacteria in the effluent in December may well be indicative of impending problems for the biological reactors.

It should be noted, however, that for bacteria present at low abundances as determined by NGS (*Nitrosomonas* and *Nitrotoga*), the FISH results, although following the same overall trend, clearly overestimate their abundance. In contrast, *Nitrospira* shows a higher relative abundance in the cDNA assay, while the FISH measurements remain consistent when median values are considered (Fig. 10). These observations support literature reports indicating that FISH provides reliable quantification when the sample contains a sufficient number of bacteria, with a detection threshold of 1·10⁶ bacteria/mL^62^.

## Conclusions

The research demonstrated the similarity between activated sludge (AS) and treated wastewater (TW) in terms of microbial composition, especially nitrifying bacteria, which may complement wastewater quality monitoring at WWTPs in the future. Using a 24-h composite sample of treated wastewater (TW) rather than a grab sample yields highly reproducible results.

The following conclusions were drawn from this work.

1. Alpha diversity analysis, based on Chao1 and Shannon indexes, showed a decrease in biodiversity in AS and TW during months with lower AS temperatures (December: 12.7ºC; February: 10.1ºC; April: 14.8ºC), and higher biodiversity in the TW than in the AS for December. The observed nitrification problems during the period of lower temperatures may have been related to the decrease in AS biodiversity and the dispersal of nitrifying species into the TW.
2. According to 16S rRNA amplicon sequencing, *Nitrospira* genus dominated the AS and TW in the periods of higher AS temperature. In the months with lower AS temperatures in the activated sludge, higher amounts of *Nitrotoga* genus were observed compared to other nitrifiers, which can be explained by the fact that they are more resistant to low temperatures. *Nitrosomonas* genus was present in every sample, with its higher amounts observed in the warmer months. In December, it was more abundant in AS than in the other nitrifiers.
3. The FISH analysis partially corresponded with the results of 16S rRNA amplicon sequencing. Most importantly, it showed a similar variation in the different groups of nitrifying bacteria, particularly the increase in these bacteria during December in the outflow. The FISH method can be used for the diagnostic analysis of suspended solids in the TW, assuming adequate bacterial abundance, as trace levels can reduce detection reliability.
4. It is advisable to continue research in this direction by collecting outflow data and subjecting it to modern statistical tools such as machine learning.

## Supplementary Information

Supplementary Information.

## Data Availability

The datasets generated during the current study are available in the NCBI SRA database repository, https://www.ncbi.nlm.nih.gov/bioproject/PRJNA1290913/, under Bioproject accession number PRJNA1290913.
