# Supplementary material for: Similarity of activated sludge and treated wastewater with special reference to nitrifiers and their seasonal variability
Source: Sci Rep. 2026 Jan 10;16:4375. doi: 10.1038/s41598-025-34503-4 (PMC12864971; doi:10.1038/s41598-025-34503-4)
Supplement: Supplementary file 1 — Supplementary Information. [file 41598_2025_34503_MOESM1_ESM.docx]

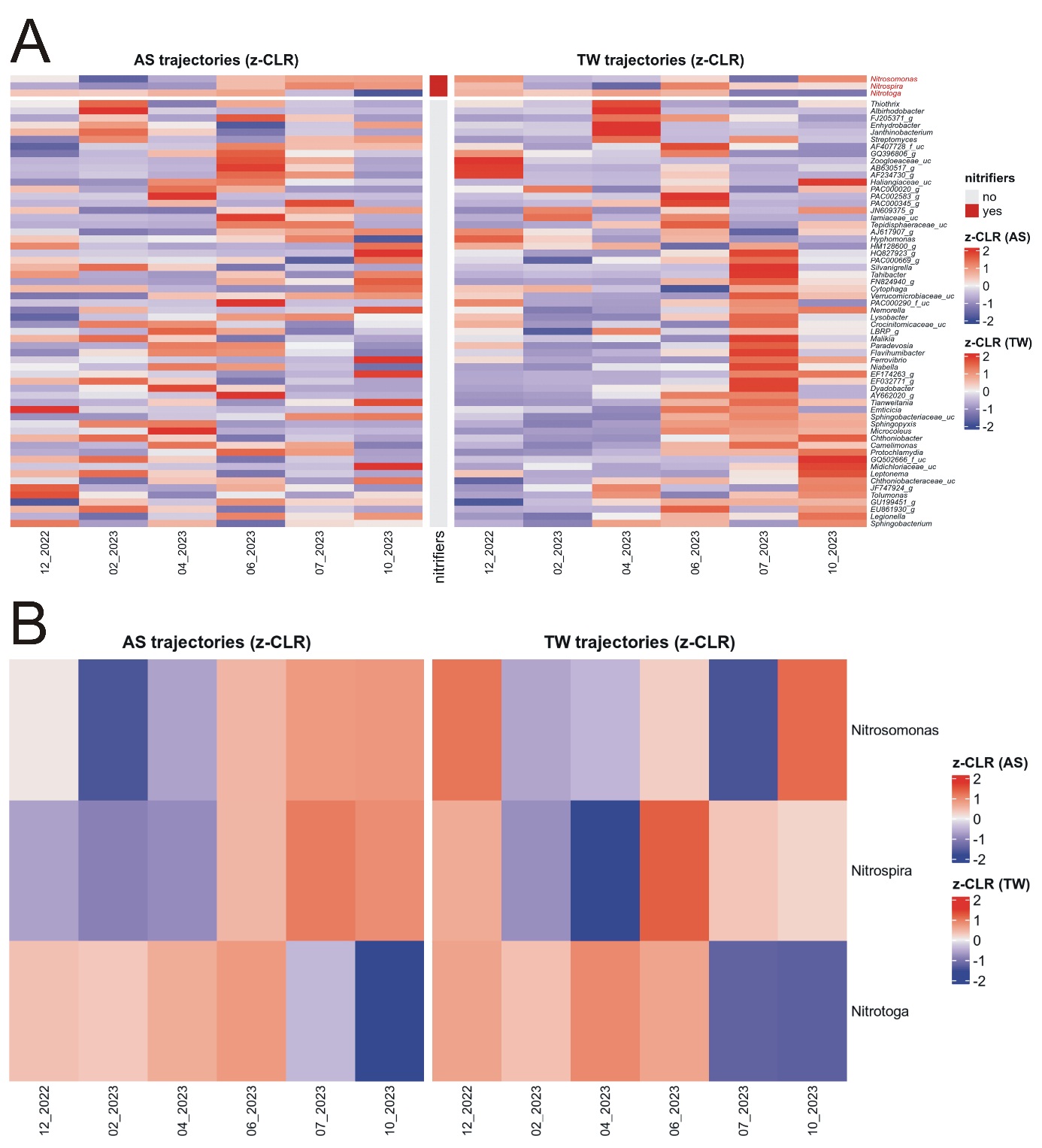


**Fig. S1. Heatmaps of genera and nitrifiers showing ΔCLR values (ΔCLR = CLR(TW) − CLR(AS)) across all sampled months.** Positive ΔCLR values (red) indicate taxa enriched in TW, whereas negative values (violet) indicate enrichment in AS. Rows (genera) are hierarchically clustered using Ward.D2 linkage, revealing clades of taxa exhibiting coherent temporal shifts between treatment conditions.
**(A)** Heatmap of the 60 most variable genera (including nitrifiers). **(B)** Heatmap restricted to detected nitrifying taxa.

Table S1. Information regarding NCBI BioProject PRJNA1290913 repository.

| **Accesion** | **BioSample** | **Sample name** |
| --- | --- | --- |
| SRR34541308 | SAMN49973841 | AC-14022023DNA |
| SRR34541307 | SAMN49973850 | TW-25072023DNA |
| SRR34541309 | SAMN49973840 | AC-181222DNA |
| SRR34541302 | SAMN49973855 | AC-13062023cDNA |
| SRR34541305 | SAMN49973852 | AC-181222cDNA |
| SRR34541301 | SAMN49973856 | AC-25072023cDNA |
| SRR34541304 | SAMN49973853 | AC-14022023cDNA |
| SRR34541300 | SAMN49973857 | AC-24102023cDNA |
| SRR34541303 | SAMN49973854 | AC-25042023cDNA |
| SRR34541306 | SAMN49973851 | TW-24102023DNA |
| SRR34541293 | SAMN49973863 | TW-24102023cDNA |
| SRR34541296 | SAMN49973860 | TW-25042023cDNA |
| SRR34541299 | SAMN49973858 | TW-181222cDNA |
| SRR34541292 | SAMN49973843 | AC-13062023DNA |
| SRR34541295 | SAMN49973861 | TW-13062023cDNA |
| SRR34541298 | SAMN49973859 | TW-14022023cDNA |
| SRR34541291 | SAMN49973844 | AC-25072023DNA |
| SRR34541294 | SAMN49973862 | TW-25072023cDNA |
| SRR34541297 | SAMN49973842 | AC-25042023DNA |
| SRR34541287 | SAMN49973848 | TW-25042023DNA |
| SRR34541290 | SAMN49973845 | AC-24102023DNA |
| SRR34541286 | SAMN49973849 | TW-13062023DNA |
| SRR34541289 | SAMN49973846 | TW-181222DNA |
| SRR34541288 | SAMN49973847 | TW-14022023DNA |

Table S2. Information regarding raw and filtered high quality read counts of amplicon samples.

| **File name** | **Raw reads** | **Filtered reads** | **High quality reads (%)** | **Q30 bases (%)** |
| --- | --- | --- | --- | --- |
| AC-13062023cDNA | 136819 | 125738 | 91.90 | 73.84 |
| AC-13062023DNA | 189889 | 170956 | 90.03 | 74.25 |
| AC-14022023cDNA | 69073 | 64139 | 92.86 | 78.14 |
| AC-14022023DNA | 65154 | 58107 | 89.18 | 77.94 |
| AC-181222cDNA | 45231 | 41114 | 90.90 | 77.34 |
| AC-181222DNA | 63411 | 56078 | 88.44 | 77.84 |
| AC-24102023cDNA | 65817 | 58176 | 88.39 | 81.21 |
| AC-24102023DNA | 86800 | 78188 | 90.08 | 81.32 |
| AC-25042023cDNA | 174829 | 154242 | 88.22 | 74.95 |
| AC-25042023DNA | 121617 | 113005 | 92.92 | 76.18 |
| AC-25072023cDNA | 182000 | 164853 | 90.58 | 71.52 |
| AC-25072023DNA | 196915 | 173159 | 87.94 | 71.97 |
| TW-13062023cDNA | 147529 | 136461 | 92.50 | 74.06 |
| TW-13062023DNA | 229497 | 209568 | 91.32 | 74.61 |
| TW-14022023cDNA | 52924 | 49622 | 93.76 | 77.43 |
| TW-14022023DNA | 58649 | 51712 | 88.17 | 78.22 |
| TW-181222cDNA | 74311 | 68402 | 92.05 | 77.51 |
| TW-181222DNA | 53736 | 47219 | 87.87 | 77.93 |
| TW-24102023cDNA | 90084 | 80603 | 89.48 | 82.13 |
| TW-24102023DNA | 98920 | 87068 | 88.02 | 81.89 |
| TW-25042023cDNA | 163001 | 148033 | 90.82 | 75.55 |
| TW-25042023DNA | 110096 | 101576 | 92.26 | 75.51 |
| TW-25072023cDNA | 228109 | 205379 | 90.04 | 72.62 |
| TW-25072023DNA | 183220 | 162750 | 88.83 | 72.35 |

Table S3. Alpha-diversity indices calculated for each sample.

| **Sample name** | **ACE*** | **Chao1** | **Jackknife** | **Shannon** | **ExpShannon (exp(H′))** | **Simpson** |
| --- | --- | --- | --- | --- | --- | --- |
| December_TW | 2854.29 | 2724.51 | 2930.39 | 6.25 | 518.01 | 0.01 |
| February_TW | 2324.10 | 2184.11 | 2352.74 | 6.03 | 415.72 | 0.01 |
| April_TW | 2838.13 | 2722.68 | 2972.00 | 6.13 | 459.44 | 0.01 |
| June_TW | 4281.12 | 4148.71 | 4462.81 | 6.67 | 788.40 | 0.00 |
| July_TW | 3570.77 | 3449.84 | 3732.68 | 6.01 | 407.48 | 0.01 |
| October_TW | 4167.80 | 4015.21 | 4352.68 | 5.99 | 399.41 | 0.02 |
| December_AS | 2641.63 | 2567.52 | 2764.40 | 6.12 | 454.86 | 0.01 |
| February_AS | 2441.09 | 2342.99 | 2489.67 | 5.99 | 399.41 | 0.01 |
| April_AS | 3184.58 | 3054.18 | 3315.85 | 6.32 | 555.57 | 0.01 |
| June_AS | 3755.60 | 3598.63 | 3926.00 | 6.7 | 812.41 | 0.00 |
| July_AS | 3411.80 | 3286.55 | 3554.65 | 6.5 | 665.14 | 0.00 |
| October_AS | 3647.59 | 3542.81 | 3796.43 | 6.44 | 626.41 | 0.01 |

*Abundance-based Coverage Estimator (ACE)
